# Supplementary material for: Conditional chemoconnectomics (cCCTomics) as a strategy for efficient and conditional targeting of chemical transmission
Source: eLife. 2024 Apr 30;12:RP91927. doi: 10.7554/eLife.91927 (PMC11060718; doi:10.7554/eLife.91927)
Supplement: Figure 3—source data 3. [file elife-91927-fig3-data3.zip › Figure 3 source data3 part 1.zip!/Figure 3-Source data 3-part1/1082-9-CRISPResso_Report_zod2ewbs.zip!/CRISPResso_on_1082_11/3b.Insertion_deletion_substitutions_size_hist.pdf]

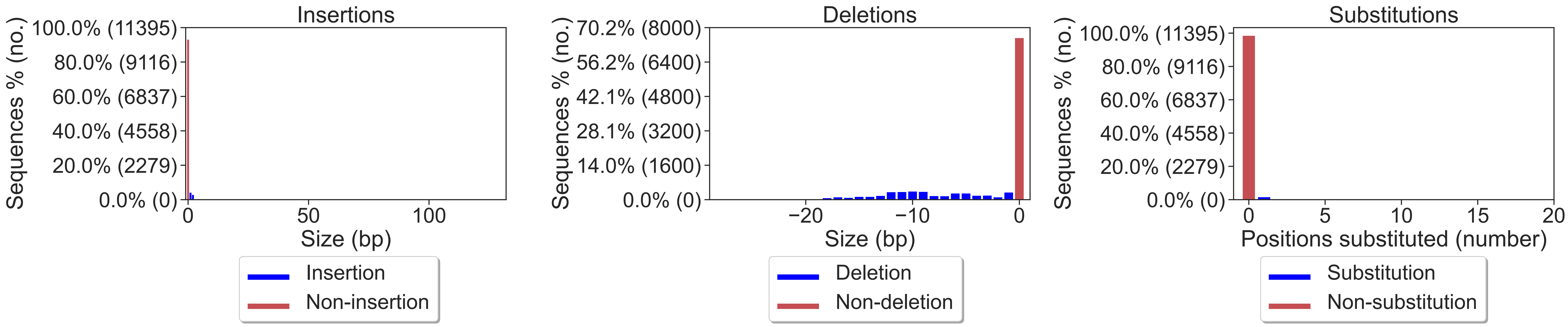
Insertions
Sequences % (no.)
100.0% (11395)
80.0% (9116)
60.0% (6837)
40.0% (4558)
20.0% (2279)
0.0% (0)
0
50
100
Size (bp)
Insertion
Non-insertion
Deletions
Sequences % (no.)
70.2% (8000)
56.2% (6400)
42.1% (4800)
28.1% (3200)
14.0% (1600)
0.0% (0)
−20
−10
0
Size (bp)
Deletion
Non-deletion
Substitutions
Sequences % (no.)
100.0% (11395)
80.0% (9116)
60.0% (6837)
40.0% (4558)
20.0% (2279)
0.0% (0)
0
5
10
15
20
Positions substituted (number)
Substitution
Non-substitution
